# Marked elevations in lung and plasma ceramide in COVID-19 linked to microvascular injury

**DOI:** 10.1172/jci.insight.156104

**Published:** 2023-05-22

**Authors:** Irina Petrache, Elisabet Pujadas, Aditya Ganju, Karina A. Serban, Alexander Borowiec, Beatrice Babbs, Irina A. Bronova, Nicholas Egersdorf, Patrick S. Hume, Khushboo Goel, William J. Janssen, Evgeny V. Berdyshev, Carlos Cordon-Cardo, Richard Kolesnick

**Affiliations:** 1 Department of Medicine, Division of Pulmonary and Critical Care, National Jewish Health, Denver, Colorado, USA.; 2 Department of Medicine, Division of Pulmonary Sciences and Critical Care Medicine, University of Colorado, Aurora, Colorado, USA.; 3 Department of Pathology, Molecular and Cell-Based Medicine, Icahn School of Medicine at Mount Sinai, New York, New York, USA.; 4 Laboratory of Signal Transduction, Sloan Kettering Institute, Memorial Sloan Kettering Cancer Center, New York, New York, USA.

**Keywords:** COVID-19, Endothelial cells

## Abstract

The pathogenesis of the marked pulmonary microvasculature injury, a distinguishing feature of COVID-19 acute respiratory distress syndrome (COVID-ARDS), remains unclear. Implicated in the pathophysiology of diverse diseases characterized by endothelial damage, including ARDS and ischemic cardiovascular disease, ceramide and in particular palmitoyl ceramide (C16:0-ceramide) may be involved in the microvascular injury in COVID-19. Using deidentified plasma and lung samples from COVID-19 patients, ceramide profiling by mass spectrometry was performed. Compared with healthy individuals, a specific 3-fold C16:0-ceramide elevation in COVID-19 patient plasma was identified. Compared with age-matched controls, autopsied lungs of individuals succumbing to COVID-ARDS displayed a massive 9-fold C16:0-ceramide elevation and exhibited a previously unrecognized microvascular ceramide-staining pattern and markedly enhanced apoptosis. In COVID-19 plasma and lungs, the C16-ceramide/C24-ceramide ratios were increased and reversed, respectively, consistent with increased risk of vascular injury. Indeed, exposure of primary human lung microvascular endothelial cell monolayers to C16:0-ceramide–rich plasma lipid extracts from COVID-19, but not healthy, individuals led to a significant decrease in endothelial barrier function. This effect was phenocopied by spiking healthy plasma lipid extracts with synthetic C16:0-ceramide and was inhibited by treatment with ceramide-neutralizing monoclonal antibody or single-chain variable fragment. These results indicate that C16:0-ceramide may be implicated in the vascular injury associated with COVID-19.

## Graphical abstract

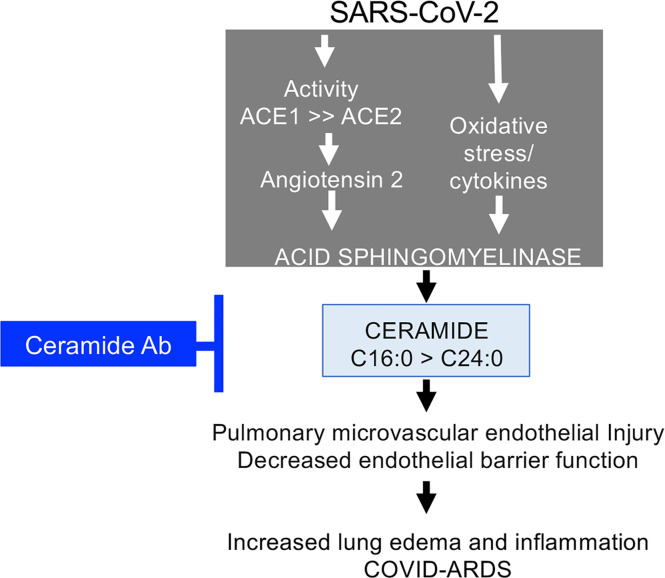

## Introduction

COVID-19–associated acute respiratory distress syndrome (COVID-ARDS) has emerged as the leading cause of death in COVID-19 (1). Although lung epithelial injury is an early event in ARDS caused by respiratory pathogens, it is rapidly followed by injury to microvascular endothelium (2). In addition to permitting the extravasation of blood components to the lung interstitium and airspaces, which impairs gas exchange, injured endothelial cells release inflammatory cytokines and microRNA-containing extracellular vesicles (3), promote local thrombosis, and intensify lung inflammation and hypoxemia. The mechanism underlying the exuberant endothelial injury that characterizes COVID-ARDS (4–8) has not been elucidated.

Disruption of sphingolipid metabolism in ARDS (9, 10) results in accumulation of ceramide, a well-established second messenger metabolite that triggers endothelial cell apoptosis during stress (11–14), including in ARDS (15, 16). With the ability to discern among different ceramide species distinguished by fatty acyl chain length and degree of saturation, specific roles of individual ceramide species are increasingly appreciated. For example, in the lung, the most abundant ceramides are palmitoyl ceramide (C16:0-ceramide; synthesized by ceramide synthase 5) and lignoceroyl ceramide (C24-ceramide; synthesized by ceramide synthase 2) (18). These 2 ceramides have distinct functions, with C16:0-ceramide being proapoptotic and C24-ceramide exerting antiapoptotic effects in lung cells (17, 18). Furthermore, data from 3 recent European cohorts and the Framingham Study (19–22) identified either an increase in C16:0-ceramide or in the ratio of C16:0-ceramide (the “bad” ceramide) to C24-ceramide (the “good” ceramide) as indicative of poor outcome of cardiovascular disease. Although targeted and nontargeted metabolomic interrogations of COVID-19 biospecimens indicate changes in various sphingolipid species in those infected with SARS-CoV-2 (23–25), a precise quantification of distinct ceramide species, their changes in the lung tissue relative to those in the systemic circulation, and their potential bioactivity as pathogenic to vascular injury and inflammation in COVID-19 are lacking.

Here, using 2 distinct COVID-19 patient cohorts, from New York City, New York (Mt. Sinai Medical Center) and Denver, Colorado (National Jewish Health), we investigate whether C16:0-ceramide might play a fundamental role in the microvascular damage associated with lethality from COVID-ARDS.

## Results

**Figure 1:**
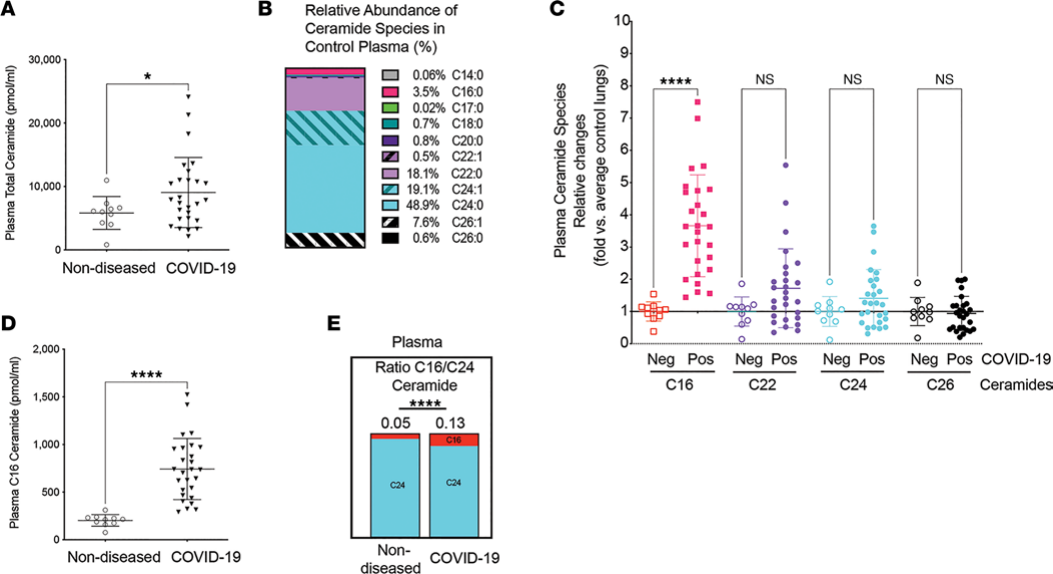
Ceramides in plasma of COVID-19 patients compared to nondiseased individuals. (**A**) Total ceramide levels measured by tandem mass spectrometry. (**B**) Relative abundance (% of total) of ceramide species in plasma of nondiseased healthy individuals. (**C**) Relative abundance (fold change) in indicated ceramide species, with horizontal black line at 1 indicating no change compared to nondiseased controls. (**D**) Absolute circulating levels of C16:0-ceramide. (**E**) Ratio of C16/C24 ceramide species. Each data point represents data from a distinct individual. Mean ± SD. *P < 0.05; ****P < 0.001 by Shapiro-Wilk test (**A** and **D**; α = 0.05, data normally distributed), unpaired *t* test with Welch’s correction (**A**, **D**, and **E**), or 1-way ANOVA with Tukey’s multiple-comparison test (**C**).

Ceramide species were measured by targeted tandem mass spectrometry (MS/MS) in the plasma of 27 patients from 2 patient cohorts with confirmed COVID-19 (Supplemental Table 1; supplemental material available online with this article; https://doi.org/10.1172/jci.insight.156104DS1), and compared to age-matched healthy controls. The total ceramide burden, composed of all species, was increased in the plasma of COVID-19 patients (Figure 1A). Consistent with published literature (26), C16-, C22-, C24-, and C26-ceramides were the most abundant ceramide species in plasma of healthy individuals (Figure 1B). Of these abundant species, C16:0-ceramide had the highest and most significant relative increase (>3-fold) in COVID-19 patient plasma compared with healthy plasma (Figure 1C). Indeed, absolute levels of circulating C16:0-ceramide increased from approximately 200 pmol/mL to approximately 750 pm/mL in COVID-19 (Figure 1D). This elevation led to a significant 2.6-fold increase in the ratio of C16-ceramide/C24-ceramide in COVID-19 plasma compared with healthy plasma (Figure 1E).

**Figure 2:**
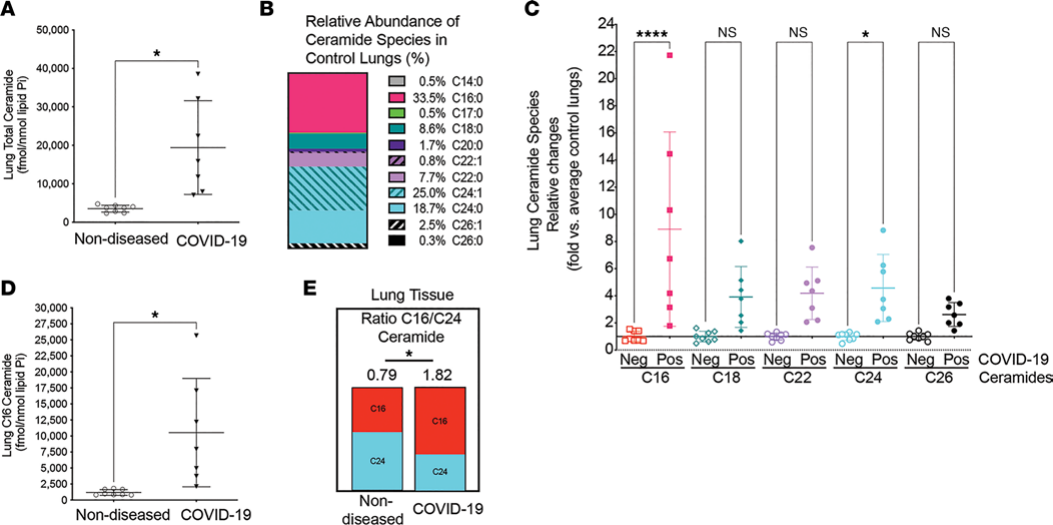
Ceramides in lungs of COVID-ARDS patients at autopsy compared to individuals without lung disease. (**A**) Total ceramide levels measured by tandem mass spectrometry. (**B**) Relative abundance (% of total) of ceramide species in lung tissue devoid of lung disease. (**C**) Relative abundance (fold change) in indicated ceramide species; horizontal black line at 1 indicates no change compared to nondiseased lungs. (**D**) Absolute levels of C16:0-ceramide. (**E**) Ratio of C16/C24 ceramide species. Each data point represents data from a distinct individual. Mean ± SD. *P < 0.05; ****P < 0.001 by Shapiro-Wilk test (**A** and **D**; α = 0.05, data normally distributed), unpaired *t* test with Welch’s correction (**A**, **D**, and **E**), or 1-way ANOVA with Tukey’s multiple-comparison test (**C**).

To investigate whether the circulating ceramide changes might reflect the lung injury that culminates in COVID-ARDS, we examined lung tissue obtained at autopsy from individuals who died of severe COVID-19. Randomly sampled lung tissue homogenates underwent lipid extraction and ceramides were measured by MS and then normalized by total lipid phosphorus. Marked increases in total ceramide were detected in COVID-19 lungs when compared with a control set of nondiseased lungs obtained from a lung donor program (Figure 2A). As previously shown (18), C16:0-ceramide and C24-ceramide are the most abundant species in nondiseased lungs (Figure 2B). While modest elevations were detected in most ceramide species from patients who died of COVID-ARDS (Figure 2C), a massive, almost 9-fold increase in C16:0-ceramide was detected (Figure 2D), leading to a reversal of the C16-ceramide/C24-ceramide ratio compared with nondiseased lungs (Figure 2E).

**Figure 3:**
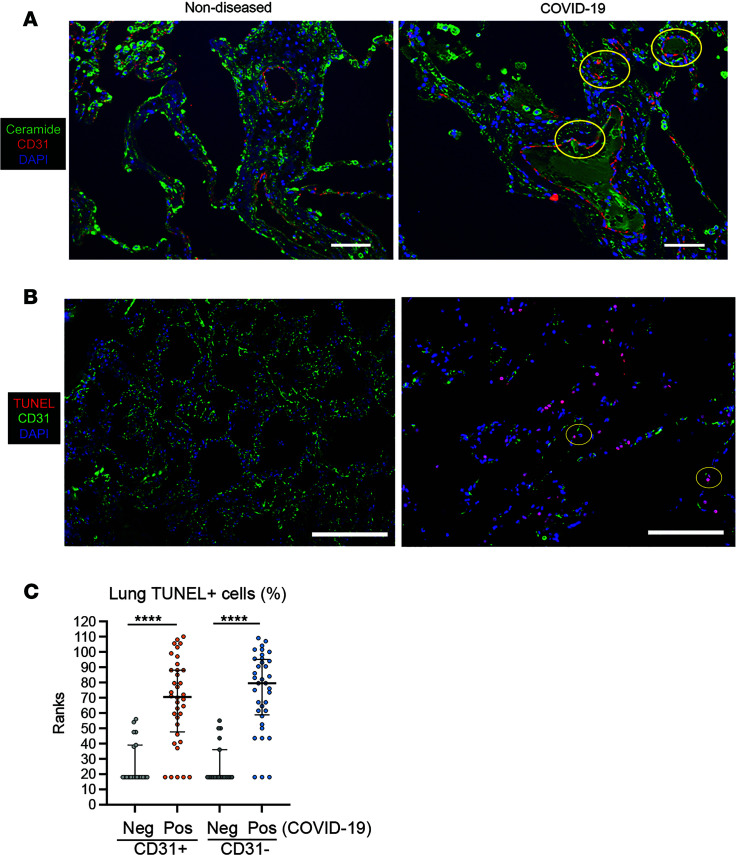
Ceramide and apoptosis in lungs of COVID-ARDS patients at autopsy compared to individuals without lung disease. Representative images of lung sections following staining with (**A**) anti-ceramide (green) and anti-CD31 (red) antibodies or (**B**) TUNEL (red) and anti-CD31 (green), all slides being costained for nuclei with DAPI (blue). Circled are areas of colocalization with vascular structures in the distal lungs. Scale bar: 50 μm (**A**) and 100 μm (**B**). (**C**) Semiquantitative unbiased assessment of TUNEL positivity in individual slides in endothelial (CD31^+^) and nonendothelial (CD31^–^) cells shown as rank-plot graph. Data analyzed nonparametrically with Kruskal-Wallis test and intergroup comparisons. ****P < 0.001.

Subsequent investigations examined the distribution of ceramide at autopsy in nondiseased and COVID-ARDS lungs by immunofluorescence microscopy using MID 15B4, a well-described anti-ceramide antibody (27). In nondiseased lungs, ceramide localization was evident predominantly in airspace-lining cells rather than vasculature-lining cells that were identified by immunostaining for the endothelial maker CD31 (Figure 3A). In contrast, in COVID-19 lungs, ceramide immunostaining was detected primarily in the lung interstitium and vascular wall, where it exhibited colocalization with CD31-positive vascular endothelial cells (Figure 3A). Consistent with this observation, COVID-19 lungs had evidence of significant injury, with a marked increase in lung parenchyma cell TUNEL staining, indicative of pervasive apoptotic death, in both nonendothelial (CD31^–^) and endothelial cells (CD31^+^) (Figure 3, B and C).

**Figure 4:**
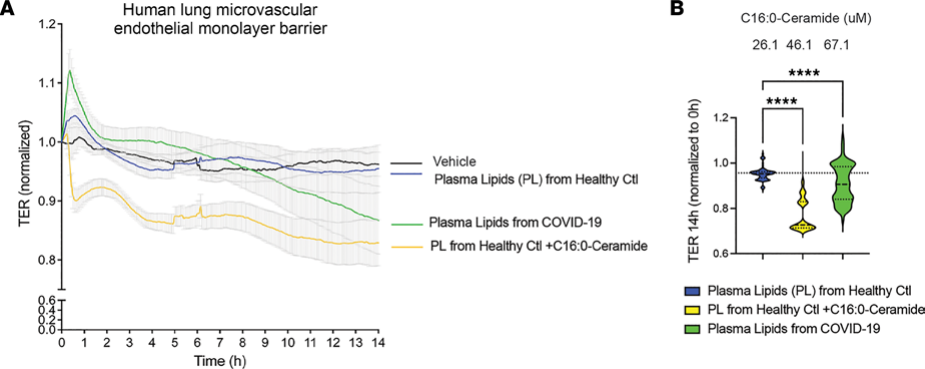
Effect of COVID-19 patient plasma lipids compared to control healthy plasma lipids on human lung microvascular barrier function. (**A**) Transcellular electrical resistance (TER) normalized to the time of initial recording (± SEM), measured over time (hours) following addition of lipids extracted from plasma of healthy individuals (Ctl PL), plasma of COVID-19 patients (COVID PL), Ctl PL supplemented with C16:0-ceramide (20 μM), or vehicle. (**B**) Violin plots of TER measured at 14 hours. Median and quartiles are shown by horizontal solid and interrupted lines, respectively. Data non-normally distributed (Kolmogorov-Smirnov). Noted above the plots are C16:0-ceramide concentrations (mean) in the respective experimental conditions, measured by mass spectrometry. ****P < 0.001 by 1-way ANOVA with Kruskal-Wallis multiple-comparison test.

**Figure 5:**
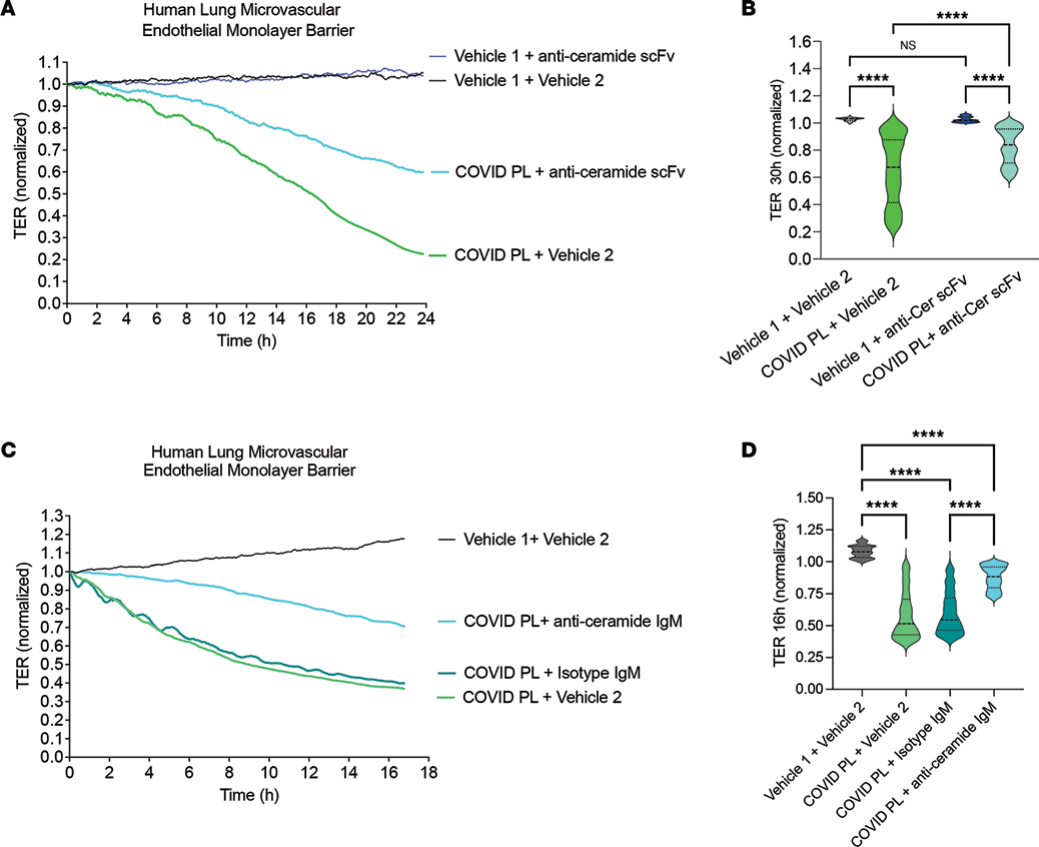
Effect of targeting plasma ceramide on lung microvascular barrier-disrupting effects of COVID-19 patient plasma lipids. (**A**–**D**) Kinetics (hours) of the transcellular electrical resistance (TER) of human lung microvascular endothelial cells following the addition of plasma of COVID-19 patients (COVID PL; pooled from *n* = 4) resuspended in vehicle 1 (solubilization buffer) coincubated with (**A** and **B**) anti-ceramide scFv (0.66 mg/mL) or vehicle 2 (PBS, 62 μL) or (**C** and **D**) immunoprecipitated with anti-ceramide IgM (667 μg/mL) or isotype IgM prior to the addition to cell monolayers. TER was normalized to baseline values (prior to onset of barrier dysfunction). (**B** and **D**) Violin plots of normalized TER at the time of maximal barrier dysfunction; median and quartiles are shown by horizontal solid and interrupted lines, respectively. Data non-normally distributed (Kolmogorov-Smirnov). ****P < 0.0001 by 2-way ANOVA and intergroup comparisons (**B**) or 1-way ANOVA with Kruskal-Wallis test and intergroup comparisons (**D**).

To explore the role of C16:0-ceramide as potential mediator of acute lung injury in COVID-19, primary human lung microvascular endothelial cells isolated from nondiseased human lungs were exposed to a lipid extract from plasma of individuals with COVID-19, followed by measurement of endothelial monolayer barrier function. Compared with lipids extracted from healthy plasma, C16-ceramide–rich plasma from COVID-19 patients caused progressive loss of barrier function (Figure 4, A and B). Exogenous addition of C16:0-ceramide to plasma lipids isolated from healthy individuals recapitulated the loss in barrier function engendered by ceramide-rich COVID-19 plasma (Figure 4, A and B). The barrier-disrupting effect of the COVID-19 plasma lipids persisted even after undergoing alkaline hydrolysis (Supplemental Figure 1), a method that deacylates glycerolipids (28), ruling out a substantive contribution of these lipids, including 1,2-diacylglycerol, to the observed effect, while preserving sphingolipids. Furthermore, treatment with 6B5 single-chain variable antibody fragment (scFv), which neutralizes ceramide as recently reported by us (29), attenuated the increase in permeability induced by COVID-19 plasma lipids (Figure 5, A and B). Similarly, immunoprecipitation of ceramides in the COVID-19 plasma lipids with a commercially available monoclonal anti-ceramide IgM markedly decreased the barrier-disrupting effects of COVID-19 plasma lipids (Figure 5, C and D). These results suggest that C16:0-ceramide elevation in COVID-19 may be pathogenic, contributing to lung microvascular injury.

## Discussion

These studies suggest that the lung microvascular injury from human coronavirus infection is, at least in part, ceramide mediated. The vascular pathogenic role of elevated C16:0-ceramide or the C16-ceramide/C24-ceramide ratio in humans has emerged from epidemiological studies of populations at risk for a major adverse cardiovascular event (MACE) (19–22). These findings support the emergence of a novel “ceramide score” for cardiovascular risk stratification that may outperform LDL cholesterol and has quickly been adopted for clinical use (30). The current study extends our understanding of the potential relevance of C16:0-ceramide to vascular disorders. While elevations in circulating C16:0-ceramide that serve as the basis of a MACE are in the range of 30% above baseline, our study identifies the largest C16:0-ceramide elevations ever reported to our knowledge, in the range of 3-fold (circulating) to 9-fold (lung autopsy data) above baseline, consistent with the severe clinical nature of COVID-ARDS. We posit that COVID-19 ARDS represents a new member of a growing list of disease states that we would like to term “ceramidopathies.”

Whereas the cardiovascular epidemiological data do not indicate whether C16:0-ceramide is a biomarker or biological mediator of vascular pathologies, a large body of work using genetic mouse models of sphingolipid signaling, including knockout mice for acid sphingomyelinase (ASMase), ceramide synthase 5, 6 (enzymes that generate C16:0-ceramide), and 2 (that generates C24-ceramides) confirm a mediator role for ceramide in various vascular pathologies (poorly controlled diabetes, atherogenesis, the gastrointestinal acute radiation syndrome), as well as in lung injury (16, 18, 31). Furthermore, studies in human lung cells and murine lungs indicate that the injurious effect of excess C16:0-ceramide balanced by the protective role of C24-ceramides is critical for determining lung cell fate and lung health (14, 17, 32).

Whereas the local elevation in lung C16:0-ceramide likely plays a principal role in outcome of lung injury, the contribution of circulating ceramide to pathophysiology is uncertain. Ceramide generated at the plasma membrane by ASMase activation serves as an evolutionarily conserved signaling mechanism that calibrates extent of cellular injury and determines cell fate in specific cell types (33). Endothelial cells are unique in having 20-fold higher ASMase expression than any other mammalian cell (34, 35), and preferentially expressing a specialized secretory form of ASMase (35), activated by diverse stresses (36). While several enzymatic pathways of sphingolipid metabolism regulate intracellular ceramide production, the circulating ceramide pool is contributed by release of ceramide-enriched extracellular vesicles from plasma membranes of various cells, an event that, during stress, involves ASMase (37). Therefore, the C16:0-ceramide elevations in COVID-19 plasma may reflect endothelial cell ASMase activation by oxidative stress (38), inflammatory cytokines (39), or excess angiotensin II (40) that characterize severe SARS-CoV-2 infection (41, 42). The subsequent activation of ASMase downstream of initial injurious responses to SARS-CoV-2 is consistent with the observation that endothelial injury follows that of the epithelium in severe COVID-19 (43).

Future studies will have to elucidate the upstream events that increase lung and plasma C16:0-ceramide and also mechanistically assess whether specific targeting of ASMase or C16:0-ceramide will mitigate acute lung injury in models of COVID-19. We also acknowledge as a limitation of our study that we did not compare the relative magnitude of injury induced by ceramide with other mediators that may induce endothelial dysfunction in COVID-19, such as exosomes, cytokines, adhesion molecules, coagulation factors, microRNAs, or nitro-oxidative stress (3).

Whether the elevation of circulating C16:0-ceramide contributes to the multiple microvascular organ syndromes widely reported to occur during severe infection with SARS-CoV-2 is currently unknown. However, extracellular ceramide incorporation into target cell membranes is recognized to trigger multiple signaling effects that recapitulate elevations of endogenous ceramide (14), including decreased lung microvascular cell barrier function (32), demonstrated here with lipid extracts from COVID-19 plasma and mimicked with exogenous C16:0-ceramide addition to healthy plasma. Furthermore, this notion is supported by nontargeted metabolomic evaluation of COVID-19 circulating extracellular vesicles, which identified a significant decrease in the C16:0-ceramide signal during the recovery phase of the disease compared with acute symptomatic COVID-19 (44).

The importance of these observations is that anti-ceramide antibodies have already been reported to be effective in treatment of multiple models of ARDS induced by acid instillation, platelet-activating factor, or lipopolysaccharide (16, 45, 46). Furthermore, an anti-ceramide scFv is in late stages of development for human use as a vascular mitigator of the lethal gastrointestinal acute radiation syndrome (29). Therefore, in addition to other proposed endothelium-protective interventions such as L-arginine (47) or antioxidants (48), targeting excess ceramide may represent a reasonable strategy to address COVID-ARDS, a disorder with high mortality that lacks effective therapy.

## Methods

### Reagents.

All reagents were purchased from Sigma-Aldrich unless otherwise stated.

Immunohistochemistry was performed on paraffin-embedded sections using the Animal Research Kit (Agilent Dako, catalog K3954) following the manufacturer’s specifications. Microscopy was performed using a Nikon Eclipse 80i inverted microscope.

### Immunofluorescence microscopy.

Lung tissue sections were probed with anti-CD31/PECAM-1 antibody (R&D Systems, catalog RB01) and anti-ceramide MID 15B4 monoclonal antibody (Enzo Life Sciences, catalog ALX-804-196-t050, lot 05301903) with secondary goat anti–mouse IgM, Alexa Fluor 488 (Invitrogen, catalog A21042). TUNEL staining was performed using the DeadEnd Fluorometric TUNEL System kit (Promega). Imaging was performed using a Zeiss LSM 700 confocal microscope with Zen Black v14 software and image processing was performed with ImageJ (NIH).

### Lipid analysis.

Methanol, water, acetonitrile (LC/MS grade), and chloroform (HPLC grade) were purchased from Thermo Fisher Scientific. *N*-acylated (C14:0-, C16:0-, C18:1-, C18:0-, C20:0-, C24:1-, C24:0-) ceramides and *N*-palmitoyl-D-erythro- sphingosine (d7-ceramide) were from Avanti Polar Lipids. The standards were dissolved in methanol and stored at –20°C.

### Lipid extraction and sample preparation for LC-MS/MS.

Lipids were extracted by modified Bligh and Dyer procedure with the use of 2% formic acid for phase separation. d7-ceramide (30 pmol) was employed as internal standard and added during the initial step of lipid extraction. Total phospholipid content was performed as described previously (49). Samples were concentrated under a stream of nitrogen, transferred into autosampler vials, and analyzed by LC-MS/MS.

Analysis of ceramides was performed by electrospray ionization tandem MS (ESI-LC/MS/MS) using a Sciex 6500 QTRAP hybrid triple quadrupole linear ion-trap mass spectrometer (AB Sciex) equipped with an Ion Drive Turbo V ionspray ionization source interfaced with a Shimadzu Nexera X2 UHPLC system. All lipid molecules and their derivatives were separated using an Ascentis Express RP-Amide 2.7 μm 2.1 × 50 mm column. Ceramides were analyzed using positive ion ESI and multiple-reaction monitoring analysis as previously described (50).

### Cell cultures.

Primary human lung microvascular endothelial cells were obtained from Lonza (CC-2527) and maintained in EBM-2 media (Lonza, CC-3156) with standard growth factor supplements EGM 2 MV Singlequots (Lonza, CC-4147) under sterile conditions at 37°C and 5% CO_2_.

### Plasma lipid extract preparation.

Plasma (500 μL) from mechanically ventilated COVID-ARDS individuals or healthy individuals was pooled from *n* = 4 to a final volume of 2 mL. An aliquot (50 μL) was used to measure C16:0-ceramide, and the remaining 350 μL of plasma was used to extract lipids. Lipids were extracted from pooled plasma using chloroform-methanol, evaporated under a stream of nitrogen gas (Organomation, N-EVAP111), resuspended in 2 mL ethanol (Thermo Fisher Scientific, BP2818-4), and stored at –20°C until used in experiments. Equal volumes (20 mL) of plasma lipids or vehicle control were added to cells, followed by measurement of barrier function. For ceramide spiking experiments, synthetic C16:0-ceramide (Avanti) was solubilized in ethanol and then resuspended in vehicle and added to cells (20 μM) simultaneously with plasma lipids. Glycerolipids were deacylated using methylamine reagent as described previously (28). After the reaction, the reagent was evaporated under the stream of nitrogen, and residual lipids were dissolved in ethanol until further processing.

Barrier function assessment was performed using electric cell–substrate impedance sensing (ECIS; Applied Biophysics) using the Z-Theta 16-well array station. ECIS 8W10E+ PET plates were coated with fibronectin (Millipore, 341631-1MG; 2.5 μg per well) for 1 hour at 37°C before cells were plated at 150,000 cells per well in 400 μL of complete (FBS-containing) EBM-2 media and cultured at 37°C with 5% CO_2_ in an ECIS incubator overnight. Cells were used in experiments once they formed monolayers, i.e., reached a capacitance of less than 10 nF at 64 kHz. Approximately 1–3 hours before treatment, media were replaced with 400 μL of serum-free EBM-2. Plasma lipids were warmed to 37°C and mixed well and 75 μL of lipids (or ethanol vehicle) was aliquoted into 1.5 mL polypropylene tubes. Following evaporation under nitrogen gas, plasma lipids were resuspended in a 75 μL resuspension buffer containing either BSA 0.1% (Figure 4) or 0.015% Tween 20 and 0.1% ethanol (Figure 5) in serum-free EBM-2 media and incubated at 37°C for 15 minutes. Samples were vortexed, placed on ice, and subjected to 4 cycles of sonication on ice until full solubilization. Electrical resistance values (Ohms) were collected at 4000 Hz over time. Plasma ceramide immune targeting was performed with anti-ceramide scFv (1.99 mg/mL, LakePharma Inc., batch TP33264F) using PBS vehicle (150 μL) as control. scFv (0.66 mg/mL) or vehicle was incubated with plasma lipids in 125 μL resuspension buffer at 37°C for 30 minutes, with initial and mid-incubation vortexing, and then mixtures were added to cell monolayers for transcellular electrical resistance (TER) recording. In separate experiments, plasma ceramide immune depletion was performed with the monoclonal anti-ceramide antibody MID 15B4 (ENZO, ALX-804-196-T050), with mouse IgM isotype (eBioscience/Thermo Fisher Scientific, 14-4752-82) as control. Anti-ceramide and control antibodies were dialyzed against PBS to remove any preservative, followed by concentration (10×) and resuspension in PBS or 0.5% BSA in PBS, respectively. Concentrated antibodies or their resuspension vehicle (100 μL) were added to plasma lipids in resuspension buffer (200 μL; final concentration 667 μg/mL) and incubated at room temperature for 45 minutes with mixing. Meanwhile, 500-μL aliquots of anti-IgM beads (Dynabeads Rat Anti-Mouse IgM, 11039D) were twice washed with solubilization buffer and then suspended in 500 μL solubilization buffer. Anti-IgM beads were then pelleted by magnet (Dynamag-2; Life Technologies, 12321D), supernatant removed, and plasma lipid antibody or control mixtures (300 μL) were added and incubated for 30 minutes at room temperature with mixing. Using the Dynamag-2, beads were separated and unbound fractions (immune depleted of ceramide) were collected and added to cell monolayers for TER recording.

### Statistics.

Analyses of significance and data normality were performed using GraphPad Prism software. A *P* value of less than 0.05 was considered statistically significant. A 2-tailed, unpaired Student’s *t* test was used for 2 groups. One-way or 2-way ANOVA testing was carried out when more than 2 variables were compared and analysis-specific post hoc tests were as stated in the figure legends.

### Study approvals.

This study was performed on deidentified human samples, being approved at the respective institution using an IRB-exempt protocol.

## Author contributions

IP, CCC, RK, and AG conceptualized the study. PSH, EP, BB, IAB, EVB, and AB developed methodology. KAS, EP, BB, NE, EVB, KG, WJJ, and AB carried out the investigation. PSH, KG, and AB generated figures. IP, WJJ, and RK acquired funding. IP and RK provided project administration. IP, EVB, WJJ, RK, and CCC supervised the study. IP and RK wrote the original draft of the manuscript, which was reviewed and edited by IP, KAS, PSH, KG, and RK.

## Supplementary Material

Supplemental data

## Version 1 05/22/2023

Electronic publication
