# Supplementary material for: Marked elevations in lung and plasma ceramide in COVID-19 linked to microvascular injury
Source: JCI Insight. 2023 May 22;8(10):e156104. doi: 10.1172/jci.insight.156104 (PMC10322682; doi:10.1172/jci.insight.156104)

Supplementary Table S1. Subjects' demographic and clinical characteristics, and investigation of biological samples assigned to each subject. Definition of smoking status: non-smoker=never smoker or not currently smoking and smoked less than 5 pack- year; ex-smoker = not currently smoking and smoked more than 5 pack- year; smoker = currently smoking.

Supplementary Table 1: Clinical Characteristics

Subjects	Clinical Information								Source	Type of Sample				Corresponding Experiments			
	Age or Age Group	Gender	COVID-19	Smoking History	Comorbidities	Clinical Manifestations at Time of Sampling	Ventilator Days	COVID-19 Treatment		HLMVEC (Passage Number)	Lung Tissue Homogenate	Plasma	Lung Tissue (Paraffin-Embedded)	Ceramide Measurements	ECIS	Immunofluorescence Ceramide	Immunofluorescence TUNEL
S1	59	F	No	ex-smoker (0.5 ppd x20-25 years)	N/A	ICH, P:F ratio 222	N/A	N/A	NJH	X (P4, 5, 6)					X		
S2	62	F	No	non-smoker	N/A	P:F ratio 310	N/A	N/A	NJH	X (P4, 5)					X		
S3	66	F	No	non-smoker	N/A	N/A	N/A	N/A	NJH	X (P4)					X		
S4	N/A	N/A	No	not available	N/A	N/A	N/A	N/A	Lonza	X (P3, 4, 5, 6)					X		
S5	58	F	No	non-smoker	N/A	P:F ratio 372	N/A	N/A	NJH		X			X			
S6	57	M	No	marijuana 2-3x/week x1 year	N/A	P:F ratio 350	N/A	N/A	NJH		X			X			
S7	52	M	No	non-smoker	N/A	P:F ratio 375	N/A	N/A	NJH		X			X			
S8	57	M	No	non-smoker	N/A	P:F ratio 362	N/A	N/A	NJH		X			X			
S9	31	M	No	1 cigarette/day x1 year	N/A	P:F ratio 491	N/A	N/A	NJH		X			X			
S10	68	F	No	non-smoker	N/A	P:F ratio 370	N/A	N/A	NJH		X			X			
S11	56	M	No	non-smoker	N/A	N/A	N/A	N/A	NJH		X			X			
S12	43	M	No	possible marijuana use	N/A	P:F ratio 435	N/A	N/A	NJH		X			X			
S13	36	F	No	N/A	N/A	N/A	N/A	N/A	NJH			X		X	X		
S14	56	F	No	N/A	N/A	N/A	N/A	N/A	NJH			X		X	X		
S15	35	F	No	N/A	N/A	N/A	N/A	N/A	NJH			X		X	X		
S16	38	F	No	non-smoker	N/A	N/A	N/A	N/A	NJH			X		X	X		
S17	37	F	No	N/A	N/A	N/A	N/A	N/A	NJH			X		X	X		
S18	32	F	No	N/A	N/A	N/A	N/A	N/A	NJH			X		X			
S19	56	F	No	N/A	N/A	N/A	N/A	N/A	NJH			X		X			
S20	37	F	No	N/A	N/A	N/A	N/A	N/A	NJH			X		X			
S21	60	F	No	N/A	N/A	N/A	N/A	N/A	NJH			X		X			
S22	35	F	No	N/A	N/A	N/A	N/A	N/A	NJH			X		X			
S23	40	F	No	N/A	N/A	N/A	N/A	N/A	NJH			X			X		
S24	79	F	No	non-smoker	N/A	P:F ratio 572	N/A	N/A	NJH				X			X	
S25	52	M	No	active smoker (30 pack-year)	N/A	P:F ratio 263	N/A	N/A	NJH				X				
S26	54	M	No	non-smoker	N/A	SAH, P:F ratio 375	N/A	N/A	NJH				X				X
S27	62	M	No	active smoker	HTN, HLD, obesity	N/A	7	N/A	NJH/SJH			X			X		
S28	62	M	No	active smoker	HTN, HLD, obesity	N/A	5	N/A	NJH/SJH			X			X		
S29	61	F	No	ex-smoker (12 pack-year)	N/A	ICH, ARDS from chest trauma, P:F ratio 115	N/A	N/A	NJH				X				X
S30	50-59	F	Yes	N/A	asthma, DM	fibrotic ARDS, infections, refractory hypoxia	N/A	HCQ, steroids	MSMC		X			X			
S31	30-39	M	Yes	N/A	HTN	AcRespFail, multiple	N/A	HCQ, steroids	MSMC		X			X			
S32	70-79	M	Yes	N/A	HTN, DM, asthma, CKD	AcRespFail requiring intubation, AKI, AFib, hypotension	N/A	anticoagulation	MSMC		X			X			
S33	70-79	M	Yes	N/A	Parkinson's, HLD, GERD	AFib, bacterial pneumonia	N/A	HCQ, anticoagulation	MSMC		X			X			
S34	60-69	M	Yes	N/A	HTN, GERD	AcRespFail with refractory hypoxia and hypotension	N/A	azithromycin, HCQ	MSMC		X		X	X		X	
S35	60-69	M	Yes	N/A	heart failure, CAD, DM, HTN, HLD, obesity	AcRespFail, gram negative sepsis	N/A	HCQ, azithromycin, anticoagulation	MSMC		X		X	X		X	
S36	60-69	M	Yes	N/A	HIV, organ transplant, HTN, cancer in remission	AcRespFail, multiple cardiac arrests	N/A	azithromycin, methylprednisolone, anticoagulation	MSMC		X		X	X		X	
S37	63	F	Yes	ex-smoker	HTN, trigeminal neuralgia, left bundle branch block, PE	N/A	4	None	NJH/SJH			X		X	X		
S38	64	M	Yes	active smoker	DM, CKD, DVT, depression, hypothyroidism, intellectual disability	N/A	5	convalescent plasma, remdesivir	NJH/SJH			X		X	X		
S39	57	F	Yes	non-smoker	DM, obesity, HTN	N/A	6	convalescent plasma	NJH/SJH			X			X		
S40	79	F	Yes	non-smoker	HTN	N/A	3	dexamethasone	NJH/SJH			X		X	X		
S41	69	F	Yes	non-smoker	hypothyroidism, DM, HTN, HLD, PE	N/A	4	none	NJH/SJH			X		X	X		
S42	53	M	Yes	non-smoker	DM, CAD, HTN, hypothyroidism, anxiety, schizophrenia, depression, OCD	N/A	2	HCQ	NJH/SJH			X		X	X		
S43	82	F	Yes	ex-smoker	HTN	N/A	1	convalescent plasma, dexamethasone	NJH/SJH			X		X	X		
S44	70	M	Yes	non-smoker	CAD, AICD placement	N/A	2	convelescent plasma, dexamethasone	NJH/SJH			X			X		
S45	40	F	Yes	non-smoker	anxiety, hepatitis, pancreatitis, alcohol abuse, essential tremor, depression	N/A	1	none	NJH/SJH			X		X	X		
S46	70	M	Yes	ex-smoker	DM, CKD, HTN, hypothyroidism, dementia	N/A	2	remdesivir	NJH/SJH			X		X	X		
S47	50	M	Yes	non-smoker	obesity	N/A	4	dexamethasone	NJH/SJH			X		X	X		
S48	82	F	Yes	ex-smoker	HTN	N/A	5	convalescent plasma, dexamethasone	NJH/SJH			X			X		
S49	56	M	Yes	ex-smoker	obesity, HTN, heart failure, obstructive sleep apnea, hypertriglyceridemia, DM	N/A	6	remdesiver, dexamethasone, convalescent plasma	NJH/SJH			X		X	X		
S50	54	M	Yes	ex-smoker	DM, obesity	N/A	8	remdesiver, dexamethasone, convalescent plasma	NJH/SJH			X			X		
S51	72	M	Yes	ex-smoker	asthma, HTN, HLD	N/A	2	convalescent plasma, remdesivir	NJH/SJH			X		X	X		
S52	56	M	Yes	non-smoker	DM	N/A	6	dexamethasone, remdesiver, convalescent plasma	NJH/SJH			X			X		
S53	50	M	Yes	non-smoker	obesity	N/A	8	dexamethasone	NJH/SJH			X			X		
S54	62	M	Yes	active smoker	HTN, ischemic stroke, alcohol and illicit substance abuse	N/A	10	none	NJH/SJH			X		X	X		
S55	50-59	F	Yes	N/A	none	died within 2 days of sampling	N/A	none	MSMC			X		X			
S56	60-69	M	Yes	N/A	leukemia in remission, sarcoidosis, DM, HLD, CAD	intubated, requiring pressors, blood and platelet transfusions, cardiac arrhythmias	N/A	enoxaparin, convalescent plasma with transfusion reaction, HCQ, steroids	MSMC			X		X			
S57	50-59	M	Yes	N/A	colon cancer	diffuse ground glass opacities, high flow nasal canula	N/A	convalescent plasma, remdesivir, enoxaparin, methylprednisolone	MSMC			X		X			
S58	30-39	M	Yes	N/A	end stage renal disease on hemodialysis, DM, cardiomyopathy, organ transplant, wound infection	required high flow nasal canula	N/A	anticoagulation	MSMC			X		X			
S59	20-29	N/A	Yes	N/A	HIV, abscess	asymptomatic COVID	N/A	none	MSMC			X		X			
S60	20-29	F	Yes	N/A	none, in labor	in labor, asymptomatic COVID	N/A	none	MSMC			X		X			
S61	60-69	M	Yes	N/A	hyperglycemia, stroke with hemiplegia, cardiac arrest, anoxic brain injury	diffuse ARDS and mild bronchiectasis, ventilator associated pneumonia, died within 2 days of sampling	N/A	none	MSMC			X		X			
S62	68	F	Yes	ex-smoker	GERD, breast cancer, HLD, osteoarthritis, preDM, OSA, stress and urinary incontinence	ARDS	4	dexamethasone, remdesivir, convalescent plasma	NJH/SJH			X		X			
S63	66	F	Yes	non-smoker	fibromyalgia	ARDS	6	Regeneron	NJH/SJH			X		X			
S64	54	M	Yes	non-smoker	alcohol abuse	ARDS	6	HCQ, methylprednisolone	NJH/SJH			X		X			
S65	89+	F	Yes	N/A	HTN, CKD, GERD	AcRespFail with refractory hypoxia and hypotension	N/A	azithromycin, HCQ, steroids, anticoagulation	MSMC				X			X	
S66	80-89	F	Yes	N/A	pancytopenia, dementia, HTN	AcRespFail, sepsis with vegetations	N/A	azithromycin, anticoagulation	MSMC				X			X	X
S67	80-89	F	Yes	N/A	HTN, DM, neurodegenerative disease	AcRespFail	N/A	azithromycin, HCQ, anticoagulation	MSMC				X			X	X
S68	60-69	M	Yes	N/A	HIV, hepatitis C with cirrhosis	AcRespFail	N/A	azithromycin, HCQ, anticoagulation	MSMC				X			X	
S69	40-49	M	Yes	N/A	HIV, HTN, asthma	AcRespFail, PE	N/A	azithromycin, HCQ, anticoagulation	MSMC				X			X	
S70	50-59	F	Yes	N/A	HTN, CAD, kidney disease	AcRespFail	N/A	azithromycin, HCQ, anticoagulation	MSMC				X			X	
S71	40-49	M	Yes	N/A	HIV, HTN, asthma	AcRespFail, PE	N/A	azithromycin, HCQ, anticoagulation	MSMC				X				X

Abbreviations: F: female; M: male; N/A: not applicable/available; NJH: National Jewish Health Human Lung Tissue Consortium, Denver, CO; SJH: Saint Joseph Hospital, Denver, CO; MSMC: Mount Sinai Medical Center, New York City, NY; X: refers to the type of sample and experiment performed; Blank: not relevant to the subject/sample; ppd: pack per day; HLMVEC: human lung microvascular endothelial cells; P: passage; ECIS: electric cell-substrate impedance sensing; P:F ratio: PaO2/FiO2 ratio; HTN: hypertension; HLD: hyperlipidemia; DM: diabetes mellitus; CAD: coronary artery disease; AKI: acute kidney injury; CKD: chronic kidney disease; OSA: obstructive sleep apnea; HIV: human immunodeficiency virus; DVT: deep vein thrombosis; PE: pulmonary embolism; SAH: subarachnoid hemorrhage; ICH: intracerebral hemorrhage; Afib: atrial fibrillation; AcRespFail: acute hypoxic respiratory failure; ARDS: acute respiratory distress syndrome; HCQ: hydroxychloroquine.

Supplementary Figure 1. Effect of hydrolyzed COVID-19 patient plasma lipids compared to unmodified COVID-19 patient plasma lipids on human lung microvascular barrier function. Transcellular electrical resistance (TER) normalized to the time of initial recording (+/- SEM), measured over time (hours) following addition of unmodified lipids extracted from plasma of COVID-19 patients (COVID PL), COVID PL following alkaline hydrolysis, or vehicle (resuspension buffer).

Supplementary Figure 1

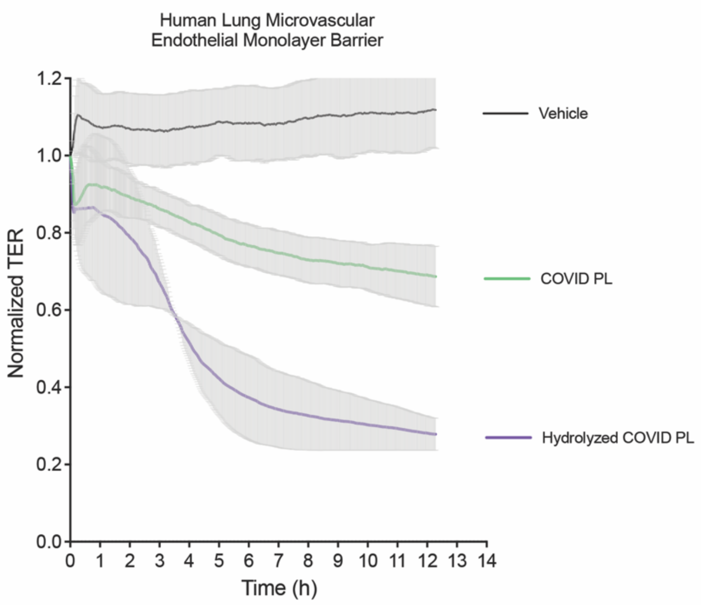

Supplement: Supplemental data [file jciinsight-8-156104-s079.pdf]
